# Endometriosis-Associated Infertility: Molecular Mechanisms and Evidence-Based Clinical Management

**DOI:** 10.3390/ijms27209040

**Published:** 2026-10-12

**Authors:** Kai-Jung Chang, Tsung-Hsuan Lai

**Affiliations:** 1 Department of Obstetrics and Gynecology, Taipei Tzu-Chi Hospital, Buddhist Tzu-Chi Medical Foundation, New Taipei City 231016, Taiwan; karen.carebear@gmail.com; 2 Division of Reproductive Endocrinology and Infertility, Department of Obstetrics and Gynecology, Cathay General Hospital, Taipei City 106438, Taiwan; 3 School of Medicine, Fu Jen Catholic University, New Taipei City 242062, Taiwan

**Keywords:** assisted reproductive technology, endometrial receptivity, immune dysregulation, ovarian endometrioma, oxidative stress, reproductive surgery

## Abstract

Endometriosis is a chronic, estrogen-dependent inflammatory disease and a major contributor to female infertility. Endometriosis-associated infertility arises from interacting anatomical and biological mechanisms, including pelvic adhesions and tubal dysfunction, reduced ovarian reserve, altered follicular and oocyte competence, oxidative stress, immune dysregulation, and impaired endometrial function. This narrative review integrates current concepts of disease pathogenesis with evidence-based fertility management. It first examines the molecular, cellular, and immune processes that support ectopic lesion establishment and may impair reproduction. It then discusses diagnostic phenotyping, infertility evaluation, prognostic tools, medical suppression, fertility-preserving surgery, intrauterine insemination, and in vitro fertilization. Particular attention is given to the strength and clinical relevance of current evidence, including surgery for ovarian endometrioma before assisted reproduction, prolonged gonadotropin-releasing hormone agonist pretreatment, fresh versus frozen embryo transfer, and the use of intracytoplasmic sperm injection without male-factor infertility. Emerging anti-angiogenic, immunomodulatory, antioxidant, stem-cell, and gene- or epigenetic-based interventions remain investigational. Because neither a single treatment nor a single assisted-reproduction protocol is optimal for all patients, management should integrate age, ovarian reserve, symptom burden, disease phenotype, prior surgery, coexisting infertility factors, and reproductive priorities. Mechanistically informed clinical trials using live birth and patient-centered outcomes are needed to advance precision fertility care.

## 1. Introduction

Endometriosis is a chronic gynecological disorder defined by endometrial-like epithelium and/or stroma outside the uterus. The disease most commonly involves the ovaries, pelvic peritoneum, uterosacral ligaments, and rectovaginal or bowel compartments. Its manifestations include dysmenorrhea, non-menstrual pelvic pain, dyspareunia, and infertility, although symptom severity correlates poorly with anatomical disease extent [1]. Endometriosis affects approximately 5–10% of reproductive-aged women, and infertility is substantially more common among affected patients than among women without the disease [1,2]. Conversely, endometriosis is identified in a considerable proportion of women undergoing evaluation for infertility [3]. These estimates vary according to the population studied, the diagnostic method, and the inclusion of asymptomatic disease.

Infertility is generally defined as failure to achieve a pregnancy after 12 months of regular, unprotected sexual intercourse. It affects approximately one in six people during their reproductive lifetime and therefore represents an important global health issue [4]. Evaluation should address both partners and should not attribute infertility to endometriosis until other common contributors, including ovulatory dysfunction, tubal disease, uterine pathology, diminished ovarian reserve, and male-factor infertility, have been considered. Endometriosis can affect several of these domains simultaneously through adhesions and anatomical distortion, ovarian endometrioma, inflammation, and altered uterine or endometrial function [3,5]. Adenomyosis frequently coexists with endometriosis and may further modify implantation and pregnancy outcomes, but it is a distinct disease rather than a subtype of endometriosis.

Endometriosis-associated infertility (EAI) is a heterogeneous condition involving multiple interacting mechanisms. Although its molecular mechanisms and reproductive management have been extensively reviewed, the extent to which individual mechanistic findings have translated into fertility-directed clinical practice remains unclear. Rather than considering molecular mechanisms and clinical management as parallel topics, this review evaluates their translational relationship by distinguishing mechanisms with established clinical implications from those that remain clinically non-actionable or investigational. Accordingly, this review aims to (1) summarize the molecular and cellular mechanisms underlying EAI; (2) examine their links to reproductive dysfunction; and (3) evaluate their current and potential implications for fertility management according to the maturity of available clinical evidence.

## 2. Review Methodology

A structured, topic-based literature search was conducted to identify evidence relevant to the pathogenesis and clinical management of endometriosis-associated infertility (EAI). PubMed was used as the primary database, and the search covered publications available through July 2026. Four predefined search domains were used: (1) mechanisms contributing to EAI, including endometrial receptivity, decidualization, oxidative stress, and immune dysfunction; (2) ovarian and tubal factors, including ovarian reserve, endometrioma, and tubal dysfunction; (3) medical and surgical management; and (4) fertility treatment using intrauterine insemination (IUI), in vitro fertilization (IVF), and assisted reproductive technology (ART). Search terms were applied to the title and abstract fields and combined using Boolean operators. Searches were restricted to English-language publications published between January 2000 and July 2026. Earlier seminal studies and additional relevant publications were identified through citation searching and the reference lists of selected reviews and guidelines.

The four PubMed searches retrieved 2159 records. After removal of 565 duplicate records based on PubMed identifiers, 1594 unique records remained for eligibility screening. Publications were considered eligible when they addressed at least one of the following review domains: (1) molecular or cellular mechanisms relevant to endometriosis and infertility; (2) ovarian, tubal, pelvic, or endometrial mechanisms contributing to reproductive dysfunction; (3) evaluation or prognostic assessment of EAI; or (4) fertility-directed medical, surgical, or assisted reproductive management. Studies of adenomyosis were considered only when they addressed its distinct but potentially coexisting contribution to infertility. Publications without direct relevance to these domains, those providing insufficient information to support the corresponding review topic, and redundant reports were excluded. Reference lists of eligible publications were additionally screened to identify relevant studies not captured by the structured database search. Following literature screening and supplementary citation searching, 105 publications were included. As this was a narrative rather than a systematic review, the final selection of studies and the decision to discuss individual studies in detail depended on author judgment based on their relevance to the review objectives and the strength of evidence levels. The included literature was therefore intended to provide a representative synthesis of the current available evidence, rather than to capture every relevant study.

Because the review encompasses heterogeneous research questions ranging from molecular mechanisms to clinical interventions, evidence was appraised according to study design, methodological rigor, directness to the corresponding review question, consistency of findings, and clinical applicability. For clinical management, evidence was hierarchically interpreted, with international evidence-based guidelines and systematic reviews or meta-analyses given the greatest weight, followed by randomized controlled trials and observational human studies. The 2022 European Society of Human Reproduction and Embryology (ESHRE) guideline on endometriosis served as the principal framework for established clinical recommendations. Mechanistic, translational, and preclinical studies were evaluated separately and were used primarily to establish biological plausibility or to discuss emerging therapeutic approaches rather than to support clinical recommendations. Lower-level evidence was considered when higher-level evidence was unavailable, particularly for emerging or insufficiently studied interventions. Where findings were inconsistent, conclusions were based preferentially on higher-level evidence, methodological quality, direct clinical relevance, and consistency across independent studies. Because of substantial heterogeneity in study populations, experimental models, interventions, and reported outcomes, quantitative synthesis was not performed Supplementary Figure S1.

## 3. Biological Basis of Endometriosis

**Figure 1:**
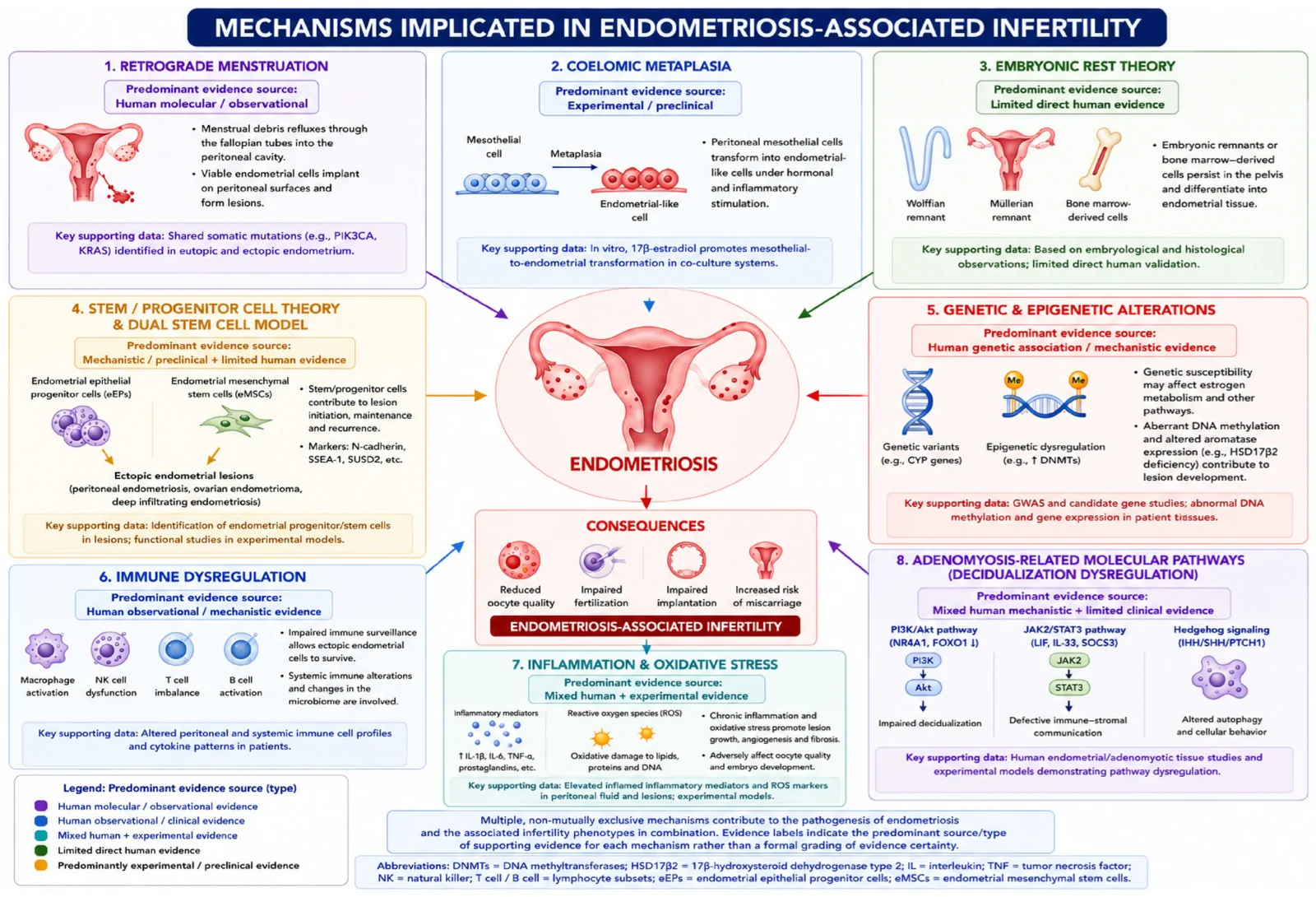
Mechanisms implicated in endometriosis-associated infertility. Retrograde menstruation, coelomic metaplasia, embryonic remnants, stem or progenitor cells, immune dysregulation, inflammation, oxidative stress, and genetic or epigenetic susceptibility may act in combination. No single mechanism explains all phenotypes. Supporting references include retrograde menstruation [7,8,9], coelomic metaplasia [10], embryonic remnants [7,8], stem/progenitor cells [8,11], genetic and epigenetic susceptibility [12,13], immune dysregulation [14,15], inflammation and oxidative stress [9,14,16], and adenomyosis-related molecular pathways [17,18,19,20,21,22,23,24]. Evidence levels indicate the predominant source and type of evidence supporting each mechanism and do not represent a formal grading of evidence certainty. Abbreviations: DNMT, DNA methyltransferase; EMSC, endometrial mesenchymal stem cell; IL, interleukin; NK, natural killer; TNF, tumor necrosis factor.

Endometriosis has three principal pelvic phenotypes: superficial peritoneal endometriosis, ovarian endometrioma, and deep endometriosis. Extrapelvic disease can involve the diaphragm, thorax, urinary tract, abdominal wall, or other sites. No single pathogenic theory explains this phenotypic diversity. Instead, lesion initiation and persistence probably reflect the interaction of cell-of-origin mechanisms with genetic susceptibility, estrogen dependence, progesterone resistance, inflammation, immune escape, angiogenesis, neurogenesis, and fibrosis Figure 1. No circulating biomarker, including cancer antigen 125 (CA-125), has sufficient accuracy for routine diagnosis [6].

### 3.1. Retrograde Menstruation and Lesion Initiation

Sampson’s retrograde-menstruation theory proposes that viable endometrial cells reach the peritoneal cavity through the fallopian tubes during menstruation, adhere to mesothelial surfaces, and establish ectopic lesions [7,8]. Molecular studies showing shared somatic mutations, including alterations in *KRAS* and *PIK3CA*, between eutopic endometrium and matched lesions support a common clonal origin for at least some disease [9]. Retrograde menstruation alone is insufficient, however, because it is common whereas endometriosis is not, and it does not fully explain distant or selected deep lesions [7,8,9]. Host susceptibility, altered cellular adhesion, impaired immune clearance, and a permissive peritoneal environment are therefore required components of this model.

### 3.2. Coelomic Metaplasia and Embryonic Remnants

The coelomic-metaplasia theory proposes that peritoneal mesothelial or related cells transform into endometrial-like tissue under hormonal, inflammatory, or other stimuli. Experimental exposure of ovarian surface epithelial and stromal cells to estradiol has produced phenotypic changes consistent with mesothelial-to-endometrial differentiation [10]. This mechanism may help explain disease in unusual clinical settings in which retrograde menstruation is implausible. A related embryonic-rest theory proposes that Müllerian or other developmental remnants persist in ectopic locations and later differentiate under endocrine or inflammatory stimulation [7,8]. These theories are complementary rather than mutually exclusive.

### 3.3. Immune Escape and Chronic Inflammation

Retrograde menstrual material would ordinarily be cleared by innate and adaptive immune responses. In endometriosis, impaired immune surveillance and altered macrophage, natural killer (NK)-cell, T-cell, and B-cell activity may permit ectopic cells to survive [14,15]. Lesion-associated macrophages can support angiogenesis, neurogenesis, fibrosis, and tissue remodeling, whereas reduced NK-cell cytotoxicity may weaken the clearance of ectopic cells. Cytokines and chemokines then sustain a positive-feedback loop among estrogen signaling, inflammation, oxidative stress, and lesion persistence. Both local peritoneal and systemic immune alterations have been described, although their causal direction and clinical utility remain incompletely defined [14,15].

### 3.4. Stem and Progenitor Cells

Endometrial epithelial and stromal progenitor cells have adhesive, proliferative, and regenerative properties that could support ectopic implantation [8]. The dual stem-cell model proposes that epithelial progenitors initiate glandular components, whereas mesenchymal or stromal progenitors support lesion expansion, fibrosis, and persistence. This framework may be particularly relevant to ovarian endometrioma and deep endometriosis [11].

### 3.5. Genetic and Epigenetic Susceptibility

Familial aggregation and genome-wide association studies support a heritable component of endometriosis. Genetic susceptibility probably modifies cellular responses to estrogen, inflammation, oxidative stress, adhesion, and tissue remodeling rather than determining disease through a single high-penetrance variant. Epigenetic changes, including altered DNA methylation, histone modification, and non-coding RNA expression, may stabilize aberrant gene-expression programs in eutopic and ectopic tissue [12,13]. These changes could also contribute to progesterone resistance and impaired decidualization. At present, genetic or epigenetic markers are not sufficiently validated for population screening, diagnosis, or treatment selection.

## 4. Clinical Phenotypes and Diagnosis Relevant to Infertility

The diagnostic approach begins with symptoms, reproductive history, physical examination, and targeted imaging. Transvaginal ultrasonography is the first-line imaging method for ovarian endometrioma and deep pelvic disease when performed by an appropriately trained examiner. Magnetic resonance imaging (MRI) is useful for mapping deep or complex disease, especially when bowel, urinary-tract, or extrapelvic involvement is suspected. A normal examination or imaging study does not exclude superficial peritoneal disease. Laparoscopy is no longer required solely to establish a diagnosis before treatment; it is reserved for situations in which surgery is clinically indicated or noninvasive findings are inconclusive and the result would change management.

### 4.1. Coexisting Adenomyosis

Adenomyosis is characterized by endometrial glands and stroma within the myometrium and commonly coexists with endometriosis. Typical manifestations include heavy menstrual bleeding, dysmenorrhea, chronic pelvic pain, uterine tenderness, and infertility. Histopathology remains definitive after hysterectomy, but clinical diagnosis increasingly relies on imaging [16,25]. Transvaginal ultrasonography is the preferred first-line modality; direct and indirect features include myometrial cysts, hyperechoic islands, fan-shaped shadowing, asymmetrical wall thickening, globular uterine configuration, and disruption or irregularity of the junctional zone. MRI can clarify equivocal cases and map diffuse or focal disease [16,26].

### 4.2. Ovarian Endometrioma

Ovarian endometrioma results from ectopic endometrial-like tissue and repeated hemorrhage within the ovary. The surrounding cortex may show inflammation, fibrosis, altered vascularity, and follicular loss, and the cyst may be densely adherent to the pelvic sidewall or adjacent organs [27,28]. On ultrasonography, a typical endometrioma is a unilocular or multilocular cyst with homogeneous low-level “ground-glass” echoes [29]. Atypical features, rapid growth, solid vascular components, papillary projections, or a postmenopausal presentation requires evaluation for alternative diagnoses, including malignancy. MRI may be useful when ultrasonographic findings are indeterminate or when comprehensive disease mapping is required.

### 4.3. Superficial Peritoneal and Deep Endometriosis

Superficial peritoneal lesions vary in appearance and are often not visible on routine imaging. Deep endometriosis is defined by infiltration beneath the peritoneal surface and may involve the uterosacral ligaments, vagina, rectovaginal septum, bowel, bladder, or ureters. Expert transvaginal ultrasonography and MRI are complementary for mapping deep lesions and anticipating surgical complexity [30,31]. The purpose of preoperative imaging is not simply to label disease but to define compartments involved, identify hydronephrosis or bowel stenosis, facilitate multidisciplinary planning, and support informed consent.

### 4.4. Epidemiological Context

A Taiwanese nationwide database analysis estimated an endometriosis prevalence of approximately 8.9%; infertility was recorded more frequently among women with endometriosis than among controls [32]. Administrative estimates should be interpreted cautiously because case definitions, diagnostic access, surgical practice, and the inclusion of asymptomatic disease influence prevalence. For clinical decision-making, the relevant question is not whether endometriosis is present in isolation, but which disease phenotype and coexisting infertility factors are most likely to limit conception.

## 5. Infertility Evaluation and Prognostic Stratification

Evaluation of EAI should follow the same couple-based principles used for all infertility. The assessment includes reproductive and medical history, age and duration of infertility, ovulatory status, transvaginal ultrasonography, tubal patency when appropriate, and semen analysis. Ovarian reserve testing with anti-Müllerian hormone (AMH) and antral follicle count (AFC) can inform counseling and treatment planning but does not directly measure oocyte quality or predict natural conception in isolation. Diagnostic testing should be individualized; routine laparoscopy, extensive endocrine panels or immunological tests are not indicated in every patient [33]. Evaluation should begin without delay when the woman is aged 35 years or older, has irregular cycles, suspected tubal disease, diminished ovarian reserve, severe endometriosis, or another condition known to impair fertility.

The revised American Society for Reproductive Medicine (rASRM) classification Table 1 describes surgical disease extent but correlates poorly with pain and has limited ability to predict pregnancy. The Endometriosis Fertility Index (EFI) Table 2 was developed to estimate the probability of non-ART pregnancy after surgery. It combines age, duration of infertility, prior pregnancy, rASRM lesion and total scores, and the postoperative least-function score of the ovaries, fimbriae, and tubes [34,35]. Higher EFI scores indicate a greater probability of natural conception. The EFI is useful for postoperative counseling, but it should not be used as a rigid threshold or as the sole basis for delaying treatment in an older patient. It also cannot be calculated reliably before surgery. Preoperative ultrasound-based prediction models are promising, particularly for deep disease, but require broader validation before replacing direct postoperative assessment [36].

## 6. Mechanisms Linking Endometriosis to Infertility

**Figure 2:**
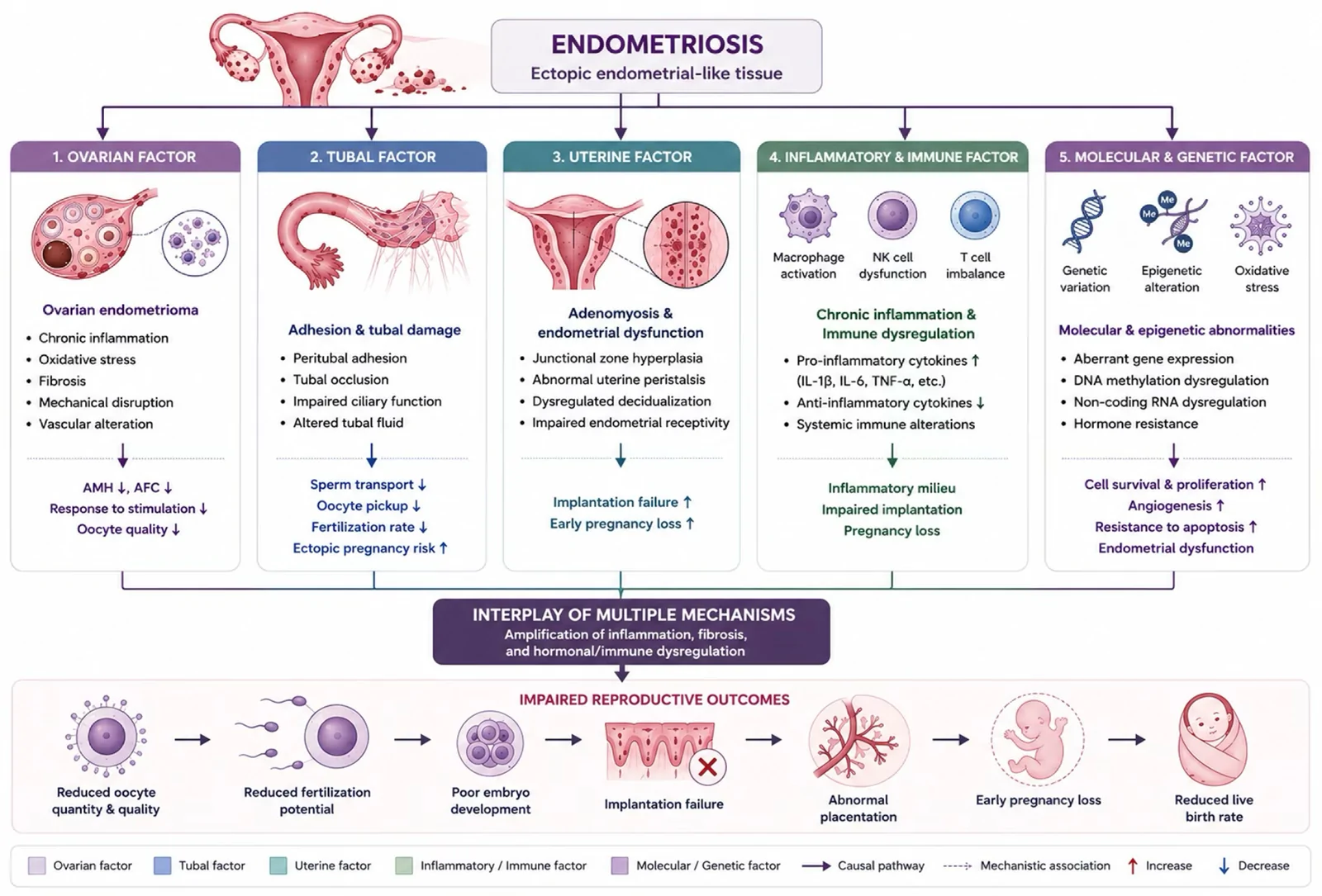
Pathogenesis of endometriosis-associated infertility. Ovarian injury, tubal and pelvic anatomical distortion, uterine or endometrial dysfunction, inflammation, immune dysregulation, oxidative stress, and molecular alterations may reduce oocyte yield, fertilization, embryo development, implantation, and live birth. Solid arrows indicate associations supported by human evidence; several molecular associations remain incompletely validated in humans. Abbreviations: AFC, antral follicle count; AMH, anti-Müllerian hormone; IL, interleukin; NK, natural killer; TNF, tumor necrosis factor.

The reproductive effects of endometriosis cannot be reduced to rASRM stage. Severe adhesions may produce an obvious mechanical barrier, whereas minimal-appearing disease may coexist with substantial inflammatory, ovarian, or endometrial dysfunction. In addition, age-related aneuploidy, polycystic ovary syndrome (PCOS), thyroid or prolactin disorders, obesity, diminished ovarian reserve, and male-factor infertility may contribute independently [38,39]. A mechanistic framework is therefore useful only when integrated with a complete couple-based evaluation Figure 2.

### 6.1. Ovarian Reserve, Folliculogenesis, and Oocyte Competence

Ovarian endometrioma is associated with lower AMH concentrations and fewer antral follicles, particularly when disease is bilateral. Proposed mechanisms include cortical stretching, local inflammation, oxidative stress, fibrosis, altered steroidogenesis, vascular injury, follicular activation followed by accelerated depletion, and granulosa cell dysfunction [40]. These associations do not prove that every untreated endometrioma causes progressive loss of functional reserve, but they support careful counseling and avoidance of unnecessary repeat ovarian surgery.

Surgery can further reduce ovarian reserve through inadvertent removal of normal cortex, thermal injury, vascular compromise, and postoperative inflammation. The magnitude of AMH decline is greater after bilateral or repeat cystectomy and may be influenced by cyst size, preoperative reserve, surgical plane, and the method of hemostasis. When technically feasible, judicious suturing or non-thermal hemostatic methods may reduce thermal damage compared with extensive bipolar coagulation [41]. AMH should be interpreted as a marker of oocyte quantity and response to stimulation, not as a direct measure of oocyte competence or the probability of natural pregnancy.

Clinical studies of IVF suggest that endometrioma may reduce the number of oocytes retrieved and increase gonadotropin requirements. A 2025 age-stratified analysis reported lower AMH and cumulative reproductive outcomes predominantly among younger women with endometrioma, whereas chronological age exerted a larger effect in older women. The number of oocytes retrieved appeared more influential than a measurable reduction in oocyte quality [42]. Residual confounding by disease severity, previous surgery, laterality, and treatment selection remains possible. The practical implication is that ovarian reserve, expected oocyte yield, prior surgery, and the accessibility of follicles should be considered together rather than using cyst diameter alone.

### 6.2. Tubal Function and Pelvic Anatomy

Normal conception requires fimbrial oocyte pickup, ciliary and muscular transport, sperm migration, fertilization, and embryo passage to the uterus. Endometriosis can impair these processes through peritubal adhesions, distortion of the tubo-ovarian relationship, intraluminal or serosal tubal disease, altered peristalsis, and inflammatory changes in tubal fluid. Tubal patency is commonly evaluated with hysterosalpingography, sonohysterosalpingography, or laparoscopic chromopertubation when surgery is otherwise indicated [43]. Treatment of tubal infertility should be individualized according to age, ovarian reserve, disease location and severity, other infertility factors, and surgical expertise. Hydrosalpinx warrants particular attention because salpingectomy or proximal tubal occlusion before IVF can improve reproductive outcomes [44].

Anti-endometrial antibodies and other circulating immune markers have been explored as indicators of tubal or endometriosis-related infertility, but none has adequate validation for routine diagnosis or treatment selection [45]. Small studies have produced inconsistent associations between rASRM stage and tubal occlusion [46]. More recent pathological observations support direct tubal involvement, particularly serosal disease in advanced endometriosis [47]. Patency alone, however, does not ensure normal fimbrial function or tubo-ovarian anatomy.

### 6.3. Uterine Transport, Endometrial Function, and Coexisting Adenomyosis

Endometriosis and adenomyosis may both impair uterine transport and endometrial function through distinct but partially overlapping mechanisms. Despite their shared pathophysiological features, they should not be considered completely interchangeable disease entities. EAI may involve altered endometrial receptivity, progesterone resistance, inflammatory signaling, and defective decidualization. In the specific context of adenomyosis, disruption of myometrial architecture and the endometrial–myometrial junctional zone may further contribute to infertility by inducing abnormal uterine peristalsis, impaired sperm transport, and increased intrauterine pressure [17,18,19]. Imaging studies have associated junctional-zone irregularity or thickening with adenomyosis, although no single thickness threshold should be used in isolation because measurements vary across the menstrual cycle and among imaging techniques [19].

Endometrial receptivity describes a transient physiological state that permits embryo attachment and invasion. Altered integrin expression, progesterone resistance, inflammatory signaling, and defective decidualization have been reported in both endometriosis and adenomyosis. Nevertheless, the relative contribution of these abnormalities may differ between the two conditions, and the clinical significance of a displaced or defective “window of implantation” is probably smaller and less uniform than previously assumed. Embryo competence remains a major determinant of implantation. Commercial receptivity tests and empiric immune or endometrial add-ons have not been shown to routinely improve live birth in women with endometriosis [48]. Molecular observations should therefore not be translated directly into unvalidated clinical interventions.

**Figure 3:**
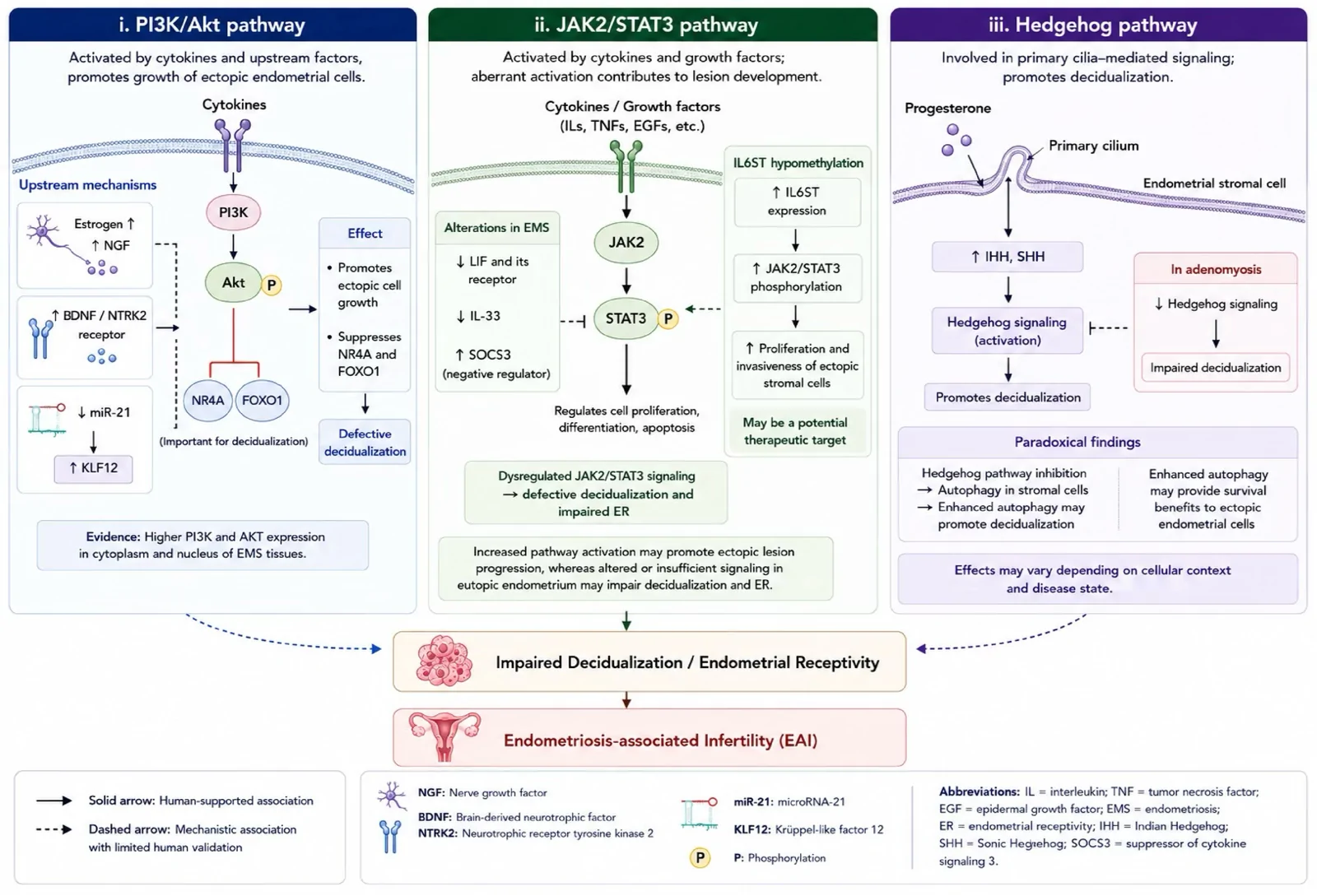
Cellular and molecular pathways potentially contributing to impaired decidualization. Dysregulated PI3K/Akt, JAK2/STAT3, and Hedgehog signaling may affect ectopic-lesion survival, eutopic endometrial function, or both. Direction and consequence depend on cell type and tissue compartment. Supporting references include PI3K/Akt signaling [21], JAK2/STAT3 signaling [20,22], and Hedgehog signaling [20,23,24]. Evidence labels indicate the predominant source/type of supporting evidence rather than a formal grading of evidence certainty. Abbreviations: BDNF, brain-derived neurotrophic factor; EGF, epidermal growth factor; ER, endometrial receptivity; FOXO1, forkhead box O1; IHH, Indian Hedgehog; IL, interleukin; KLF12, Krüppel-like factor 12; LIF, leukemia inhibitory factor; NGF, nerve growth factor; NTRK2, neurotrophic receptor tyrosine kinase 2; SHH, Sonic Hedgehog; SOCS3, suppressor of cytokine signaling 3; TNF, tumor necrosis factor.

Decidualization of endometrial stromal cells depends on coordinated progesterone-responsive transcription, intracellular signaling, metabolic remodeling, and immune–stromal communication. Several molecular pathways implicated in impaired decidualization have been particularly investigated in adenomyosis-associated infertility [20]. Given the partially overlapping biological features of adenomyosis and endometriosis, these pathways may also provide insights into endometrial dysfunction in patients with endometriosis, although their relevance to EAI remains to be fully established. Importantly, these pathways should also be interpreted according to tissue compartment, as signaling that promotes the survival of ectopic lesions may exert different effects in the eutopic endometrium (Figure 3).

#### 6.3.1. PI3K/Akt Signaling

Activation of phosphoinositide 3-kinase (PI3K) and Akt promotes cellular survival and proliferation. In ectopic tissues, dysregulated PI3K/Akt signaling has been implicated in impaired decidualization through suppression of decidualization-related transcription factors, including forkhead box O1 (FOXO1) and nuclear receptor subfamily 4 group A members. Proposed upstream regulators include nerve growth factor, brain-derived neurotrophic factor/neurotrophic receptor tyrosine kinase 2, Krüppel-like factor 12, and microRNA-21 [20]. Separately, increased PI3K and Akt expression has also been demonstrated in endometriotic tissue [21], where pathway activation may contribute to inflammation and ectopic-lesion survival. These observations should not be interpreted as evidence that PI3K/Akt dysregulation has identical reproductive effects in endometriosis and adenomyosis.

#### 6.3.2. JAK2/STAT3 Signaling

Janus kinase 2/signal transducer and activator of transcription 3 (JAK2/STAT3) signaling integrates interleukin, growth-factor, and other cytokine signals. In adenomyosis, alterations involving leukemia inhibitory factor, interleukin-33, suppressor of cytokine signaling 3, and related JAK2/STAT3 signaling have been implicated in defective decidualization and impaired endometrial function [20]. In endometriosis, hypomethylation and overexpression of IL6ST have separately been linked to JAK2/STAT3 activation in experimental models [22]. Although these findings arise from different disease and tissue contexts and should not be considered equivalent evidence for infertility, they may suggest that dysregulation of the JAK2/STAT3 pathway represents a shared molecular mechanism potentially contributing to infertility in both endometriosis and adenomyosis.

#### 6.3.3. Hedgehog Signaling and Autophagy

Primary cilia and Hedgehog signaling participate in progesterone-responsive stromal-cell decidualization. Altered Hedgehog signaling has been implicated in adenomyosis-associated endometrial dysfunction [20], while experimental models have also demonstrated an important role for Hedgehog signaling in normal endometrial remodeling and myometrial homeostasis [23]. Hedgehog inhibition and enhanced autophagy have additionally been reported in both endometriosis and adenomyosis [24]. These pathways are mechanistically attractive but are not validated targets for fertility treatment.

### 6.4. Adhesions and Fibrosis

Pelvic adhesions may arise from endometriosis itself or from previous surgery. Mesothelial injury, fibrin deposition, impaired fibrinolysis, macrophage activation, and fibroblast-to-myofibroblast differentiation contribute to their formation. Adhesions can immobilize the ovary, separate the fimbriae from the ovulatory surface, obstruct the tube, and complicate oocyte retrieval or subsequent surgery [49,50]. The relationship between adhesions and implantation is less direct than their mechanical effect on gamete transport. Meticulous tissue handling and minimization of ischemic or thermal injury remain central to prevention; evidence for many barrier agents is limited.

### 6.5. Inflammation, Immune Dysregulation, Oxidative Stress, and the Microbiome

Peritoneal fluid from women with endometriosis may contain increased concentrations of interleukin-1beta, interleukin-6, tumor necrosis factor-alpha, prostaglandins, chemokines, reactive oxygen species, and activated macrophages. This milieu can affect sperm function, folliculogenesis, oocyte maturation, fertilization, and embryo development in experimental systems. Iron released by repeated hemorrhage can catalyze oxidative reactions, damage lipids and DNA, and reinforce inflammation and fibrosis [51]. These findings provide biological plausibility, but biomarker heterogeneity and overlap with unaffected populations have limited clinical translation.

Altered NK-cell cytotoxicity and T-cell, B-cell, and macrophage phenotypes may promote immune tolerance of ectopic tissue [51]. Endometrial or reproductive-tract microbiome differences and chronic endometritis have also been reported in association with endometriosis [52]. It remains uncertain whether these are causal, secondary to inflammation or treatment, or partly explained by sampling and laboratory methods. Routine microbiome testing, endometrial biopsy for plasma cells, cytokine profiling, or empiric antimicrobial and immunological treatment cannot be recommended solely because a patient has endometriosis.

### 6.6. Genetic and Epigenetic Contribution to Infertility

Susceptibility variants and epigenetic alterations can influence estrogen metabolism, inflammatory responses, progesterone signaling, angiogenesis, and fibrosis. Increased DNA methyltransferase expression and locus-specific methylation abnormalities have been described in ectopic tissue [17]. However, evidence that any specific variant or epigenetic signature independently predicts infertility, ART response, or live birth is insufficient. Molecular stratification remains a research objective rather than a current clinical test.

The mechanisms underlying EAI vary considerably in their degree of clinical translation. Ovarian reserve impairment, tubo-ovarian distortion, and pelvic adhesions already have direct implications for fertility assessment and decisions regarding surgery or ART. In contrast, inflammatory, immune, oxidative, endometrial, and epigenetic abnormalities provide important mechanistic insights, but their direct impact on treatment selection remains less well established. Although molecular targets have emerged as potential therapeutic strategies, most are still supported predominantly by preclinical or limited clinical evidence [53]. Taken together, this reveals an important translational imbalance in EAI: mechanisms that produce measurable structural or reproductive consequences can already inform clinical decisions, whereas increasingly characterized molecular abnormalities have not yet generated comparably actionable tools for patient stratification or treatment selection. The major translational gap may therefore lie not in identifying additional molecular pathways, but in determining whether a specific mechanism contributes meaningfully to infertility in an individual patient and predicts response to a fertility-directed intervention. Future translational studies should consequently prioritize validation of mechanism-based biomarkers against clinically meaningful reproductive outcomes, particularly treatment response and live birth. The following section discusses current and emerging management strategies for EAI in the context of available evidence.

## 7. Evidence-Based Clinical Management

Management should begin with the patient’s immediate goal. Hormonal suppression is appropriate for pain or disease control when pregnancy is not currently desired, but it prevents or delays conception during treatment and does not improve natural pregnancy rates by itself. Fertility-directed options include expectant management after appropriate evaluation, surgery in selected patients, IUI with ovarian stimulation, and IVF. Age, ovarian reserve, duration of infertility, symptom burden, disease phenotype, prior surgery, tubal status, semen findings, and patient preference should be considered together. Importantly, the clinical relevance of the mechanisms discussed above is unequal: anatomical distortion and ovarian reserve impairment can directly influence treatment selection, whereas most molecular and inflammatory abnormalities have not yet yielded validated biomarkers or mechanism-specific fertility treatments.

The evidence supporting different management strategies varies considerably in quality and maturity. Throughout this section, the 2022 ESHRE guideline serves as a reference framework for established clinical recommendations, supplemented by systematic reviews, randomized trials, and observational studies where appropriate. For strategies lacking higher-level evidence, smaller studies and limited reports are still included to reflect the available literature, with their evidentiary limitations acknowledged.

### 7.1. Medical Therapy: Symptom Control Versus Fertility Treatment

Combined hormonal contraceptives and progestins are established treatments for endometriosis-associated pain [54,55,56]. They reduce ovulation or endometrial proliferation, menstrual flow, and estrogen-dependent inflammatory activity. Continuous regimens may provide better suppression of dysmenorrhea and postoperative symptom recurrence than cyclic use in selected patients [54]. It is important to note that ovarian suppression should not be prescribed to increase spontaneous pregnancy in women with EAI actively attempting conception [54].

Combined hormonal contraceptives are reversible and do not cause permanent loss of ovarian reserve, although pregnancy is not expected while they are used [57]. Progestin options include dienogest, norethisterone acetate, depot formulations, implants, and the levonorgestrel-releasing intrauterine system. Their systemic and contraceptive effects differ; notably, dienogest prescribed for endometriosis should not automatically be considered a reliable contraceptive. Breakthrough bleeding, mood symptoms, and metabolic or bone effects vary by agent. Postoperative hormonal suppression should not be prescribed solely to enhance future pregnancy rates; however, it may be offered to women with no immediate pregnancy plans for symptom control without adversely affecting future fertility [54,55,56].

The 2022 ESHRE guideline considers the evidence insufficient to recommend routine prolonged combined hormonal contraceptive or progestogen pretreatment before ART to improve live birth rates [54].

#### 7.1.1. GnRH Agonists and Antagonists

Continuous gonadotropin-releasing hormone (GnRH) agonist exposure causes initial stimulation followed by pituitary receptor downregulation and profound ovarian suppression. GnRH agonists are effective for pain but can cause vasomotor symptoms, urogenital symptoms, mood changes, and bone loss; add-back therapy reduces hypoestrogenic adverse effects during longer courses [55,56]. Extended GnRH agonist pretreatment before IVF was historically proposed to reduce inflammatory disease activity. More recent systematic reviews and network meta-analyses have not shown a consistent improvement in live birth or clinical pregnancy over standard IVF protocols [58]. Similarly, evidence does not support routine GnRH agonist pretreatment before hormone-replacement frozen embryo transfer in all women with endometriosis [59]. The 2022 ESHRE guideline does not recommend extended GnRH agonist administration before ART to improve live birth due to uncertain benefits [54].

Oral GnRH antagonists suppress gonadotropin secretion rapidly and without an initial flare. Their dose-dependent hypoestrogenic effects and the need for add-back therapy at higher doses resemble those of GnRH agonists. They are approved in some jurisdictions for endometriosis-associated pain, not as established fertility-enhancing pretreatment. The PREGNANT trial protocol was designed to test whether eight weeks of elagolix before IVF improves live birth, but a protocol alone does not provide efficacy evidence [60]. Until adequately powered results are available, GnRH antagonist pretreatment should be considered investigational for improving IVF outcomes.

#### 7.1.2. Other Hormonal Options

Aromatase inhibitors such as letrozole or anastrozole can reduce estrogen synthesis and may be used off-label for refractory pain in combination with ovarian-suppressive therapy, as recommended by the 2022 ESHRE guideline [54]. Their adverse effects include bone loss, vasomotor symptoms, and functional ovarian cysts. Evidence is insufficient to recommend aromatase-inhibitor cotreatment routinely to improve fertility or IVF outcomes in EAI. Selective progesterone-receptor modulators remain limited by uncertain long-term endometrial and hepatic safety and should not be used as fertility treatment outside research settings [61].

### 7.2. Emerging Mechanism-Based Therapies

The biological heterogeneity of endometriosis has stimulated interest in non-hormonal approaches that could suppress lesions without preventing ovulation. These approaches provide a potential link between emerging molecular insights and fertility-directed management; however, most remain supported by limited clinical or preclinical evidence so far.

#### 7.2.1. Anti-Angiogenic Therapy

Endometriotic lesions require vascular support, and vascular endothelial growth factor (VEGF) is increased in many experimental and clinical samples [55,62]. Dopamine agonists and other anti-angiogenic agents can reduce lesion vascularity in animal models; cabergoline and dienogest have shown additive effects in preclinical experiments [63]. A single human case involving bevacizumab demonstrated possible benefits on endometriosis-associated symptoms and ovarian reserve markers, but is not sufficient to establish efficacy, ovarian benefit, or reproductive safety [64]. A recent systematic review and network meta-analysis confirmed that evidence for VEGF-directed therapies remains predominantly preclinical, with mostly short-term and heterogeneous beneficial effects reported in rodent models and limited human data [65]. Because angiogenesis is essential for folliculogenesis, implantation, placentation, and fetal development, systemic anti-angiogenic therapy should not be used to treat EAI outside rigorously designed research.

#### 7.2.2. Immunomodulatory Therapy

Proposed targets include tumor necrosis factor, CD47-signal regulatory protein alpha signaling, NK-cell function, macrophage polarization, ferroptosis, and microRNA-mediated immune regulation. Most supporting evidence is preclinical, and anti-tumor-necrosis-factor therapy has not consistently improved pain or lesion outcomes in clinical studies [66,67]. A retrospective cohort reported higher implantation and clinical pregnancy rates after peri-implantation adalimumab but no statistically significant improvement in ongoing pregnancy or live birth [68]. Given the observational design, potential cointerventions, and limited evidence for reproductive benefit, routine immunomodulatory therapy cannot currently be recommended for EAI.

#### 7.2.3. Antioxidants and Metabolic Interventions

Vitamins C and E, N-acetylcysteine, zinc, metformin, statins, and related agents have shown antioxidant or lesion-modifying effects in experimental studies [55,69]. A 2025 systematic review and meta-analysis of 21 RCTs found improvements in selected pain and oxidative-stress outcomes, but no significant improvement in clinical pregnancy rate [70]. Heterogeneity in formulations, doses, study populations, and outcomes limits inference. Nutritional adequacy and treatment of documented deficiencies are appropriate, but antioxidant supplements should not replace established fertility treatment.

#### 7.2.4. Stem-Cell, Secretome, Gene, and Epigenetic Approaches

Mesenchymal stem cells and their secretomes have immunomodulatory, anti-inflammatory, and regenerative properties, but they may also interact unpredictably with lesion growth [66,71]. Current evidence remains predominantly preclinical, with a recent systematic review reporting generally favorable effects but substantial heterogeneity across experimental models, cell types, and outcomes [71]. In a murine model, adipose-derived stem-cell conditioned medium improved markers of endometrial receptivity and several pregnancy outcomes [72]; translation to human fertility requires careful evaluation of cell source, biodistribution, tumorigenicity, immunogenicity, and offspring safety. Gene- and epigenetic-based approaches targeting VEGF, CD44, autophagy, non-coding RNA, DNA methylation, or histone deacetylation are similarly experimental. They should be framed as mechanistic research directions, not near-term clinical alternatives.

### 7.3. Fertility-Preserving Surgery

Surgery may relieve pain, restore anatomy, obtain histological diagnosis when needed, treat organ-threatening disease, and create an opportunity for natural conception. It can also delay ART, generate adhesions, diminish ovarian reserve, and introduce perioperative and obstetric risks. Therefore, surgical decisions should be individualized based on age, symptoms, patient preference, gynecologic and surgical history, and the EFI [54].

The 2022 ESHRE guideline suggests that operative laparoscopy may improve ongoing pregnancy in women with rASRM stage I/II endometriosis with weak evidence. Routine surgery before ART, however, is not suggested owing to unclear benefits [54].

#### 7.3.1. Coexisting Adenomyosis

The ESHRE does not consider adenomyosis as a subgroup of endometriosis [54]. Hence, management of coexisting adenomyosis should be considered separately from the surgical management of endometriosis because of their distinct anatomical involvement and reproductive implications. Hysterectomy is definitive treatment for adenomyosis when fertility is no longer desired. For selected patients seeking pregnancy, uterus-sparing adenomyomectomy or focal excision may reduce symptoms and reconstruct the uterine wall, but evidence for improved live birth is based mainly on heterogeneous observational studies. Diffuse disease may be difficult to excise completely, and extensive reconstruction can weaken the myometrium. Reported subsequent risks include uterine rupture, preterm birth, placenta previa, and placenta accreta spectrum [73].

Uterine artery embolization reduces lesion perfusion but has uncertain effects on ovarian reserve, implantation, and pregnancy; it is not a routine fertility treatment [74]. High-intensity focused ultrasound and radiofrequency ablation are uterus-sparing options for selected symptomatic patients, but reproductive evidence is limited and selection criteria are not standardized [75]. Reviews of conservative adenomyosis surgery report pregnancies and live births but substantial heterogeneity in phenotype, technique, adjunctive suppression, and follow-up [73,76]. No universal operation or mandatory interval to conception has been established. Preconception imaging of the residual myometrium and individualized obstetric planning are prudent after major reconstruction.

#### 7.3.2. Ovarian Endometrioma

Routine surgery for an ovarian endometrioma before ART is not recommended solely to improve live birth, because benefit has not been demonstrated and surgery can reduce ovarian reserve [54]. Indications may include significant pain, suspicious or atypical imaging, rapid growth, inability to access follicles safely, risk of rupture or infection in a selected case, or a patient preference for surgery after informed counseling. Decision-making should consider age, AMH and AFC, laterality, cyst size and appearance, previous ovarian surgery, symptom burden, other infertility factors, and the planned time to pregnancy [77]. A diameter threshold alone is insufficient. This illustrates a relatively direct mechanism-to-management relationship in EAI: because both endometrioma-associated ovarian injury and surgical treatment may compromise the follicular pool, evidence of impaired ovarian reserve argues for greater caution toward repeat or non-essential ovarian surgery rather than more aggressive lesion removal.

The surgical approach should aim to minimize ovarian damage while reducing the risk of recurrence. ESHRE recommends cystectomy over drainage and coagulation, while CO_2_ laser vaporization may also be considered [54]. Ablative approaches using CO_2_ laser or plasma energy may preserve more ovarian tissue in selected cases, although comparative data on live birth and long-term recurrence remain limited [78]. The surgeon should identify the correct tissue plane, minimize traction and thermal spread, and avoid unnecessary repeat procedures. The decline in AMH is often greater after bilateral than unilateral cystectomy and may persist beyond the immediate postoperative period [79]. AFC and AMH should be interpreted together, and fertility preservation may be discussed before bilateral, recurrent, or extensive ovarian surgery.

No evidence-based fixed interval between endometrioma surgery and ART applies to all patients. Once recovery is adequate, timing should be determined by age, ovarian reserve, postoperative anatomy, and whether spontaneous conception is reasonable. A wide interval reported in an observational study should not be converted into a universal recommendation [80].

#### 7.3.3. Tubal Disease and Hydrosalpinx

A laparoscopic cohort found higher rates of tubal occlusion among women with EAI than among infertile controls, with occlusion associated with more advanced rASRM stage and secondary infertility [81]. Management depends on the site and severity of disease. Mild distal disease may be considered for fimbrioplasty or neosalpingostomy in a young patient with good ovarian reserve and no major additional infertility factor, whereas severe hydrosalpinx generally favors salpingectomy or proximal occlusion followed by IVF. Tubal reconstructive surgery carries a risk of ectopic pregnancy and may be less appropriate when age or ovarian reserve makes delay consequential [82]. This provides another direct mechanism-to-management link: when infertility is predominantly related to surgically correctable tubal distortion, reconstruction may restore the anatomical pathway required for natural conception, whereas IVF bypasses impaired gamete transport when tubal damage is severe or unlikely to be functionally reversible.

Recent international guidance emphasizes individualized selection between tubal surgery and IVF. Surgery may be reasonable for women younger than 35 years with mild-to-moderate, surgically correctable tubal disease, whereas IVF is generally favored for severe disease and for older patients. The certainty of comparative evidence is low, and decisions should also incorporate semen findings, ovarian reserve, surgical expertise, cost, access, and patient values [83].

#### 7.3.4. Adhesiolysis and Adhesion Prevention

Adhesiolysis may restore tubo-ovarian relationships when adhesions are clinically important, but recurrence and organ injury are relevant risks. MRI can identify dense bands, organ tethering, or anatomical distortion and can assist preoperative planning, although it has limited sensitivity for fine adhesions and cannot replace surgical assessment [84]. Prevention depends primarily on gentle tissue handling, meticulous hemostasis, limited desiccation and thermal injury, and reduction of foreign material. Selected barrier agents may reduce postoperative adhesion formation, but evidence that they improve live birth is sparse [85,86].

### 7.4. Assisted Reproductive Technology

Assisted reproductive technology (ART) bypasses several anatomical barriers created by endometriosis. The 2022 ESHRE guideline supports ART for EAI, particularly when tubal function is compromised, male factor infertility coexists, the EFI is low, or other treatments have failed. ART should not be withheld because of concerns that ovarian stimulation may accelerate disease, as ESHRE considers ART safe with respect to endometriosis recurrence [54], with evidence likewise showing no clinically important increase in recurrence [87,88]. Treatment should not be unnecessarily delayed when maternal age, diminished ovarian reserve, or prolonged infertility makes spontaneous conception unlikely. Thus, the rationale for ART in EAI is strongest when the dominant reproductive impairment is one that ART can directly bypass, particularly tubo-ovarian distortion or compromised gamete transport. In contrast, proposed molecular abnormalities in endometrial receptivity, inflammation, or immune function currently provide little basis for selecting a specific ART strategy because validated markers linking these mechanisms to treatment response are lacking.

#### 7.4.1. Intrauterine Insemination

IUI with ovarian stimulation can be considered in women with rASRM stage I/II endometriosis, while its value in stage III/IV disease with tubal patency is less certain but may still be considered [54]. Mild stimulation is intended to increase the number of available oocytes while limiting multiple pregnancy and ovarian hyperstimulation syndrome (OHSS). Per-cycle success depends mainly on age, duration of infertility, tubal function, follicular response, and semen quality. Repeated low-probability cycles should be avoided when time is clinically important; three to four stimulated cycles are a reasonable maximum for many patients before reassessment, although the exact number should be individualized [89].

Routine IUI add-ons have limited evidence. Luteal progesterone may be considered after gonadotropin stimulation, but endometrial scratching, double insemination, prolonged bed rest, nonstandard catheter techniques, and empiric changes in sperm preparation have not consistently improved live birth [90]. In women with more advanced disease, IUI may still be considered when tubal patency and other prognostic factors are favorable, although IVF is generally more efficient when tubal distortion, older age, diminished ovarian reserve, or other infertility factors are present [91].

#### 7.4.2. In Vitro Fertilization

IVF is appropriate for advanced endometriosis, bilateral tubal compromise, significant male factor, diminished ovarian reserve, low postoperative fertility prognosis, failure of expectant management or IUI, or a need to minimize time to pregnancy [54]. Oocyte retrieval and fertilization bypass tubo-ovarian distortion, but ovarian endometrioma, prior surgery, and reduced reserve may lower oocyte yield [92]. Counseling should emphasize cumulative live birth rather than the outcome of a single transfer.

The risks associated with IVF include OHSS, procedure-related complications, multiple pregnancy when more than one embryo is transferred, and differences in selected obstetric outcomes. Many observed risks are partly attributable to parental age, infertility diagnosis, comorbidity, and multiple gestation rather than the laboratory procedure alone [93,94]. Single-embryo transfer and contemporary OHSS-prevention strategies substantially reduce avoidable risk.

##### Ovarian Stimulation Protocols

No specific ovarian stimulation protocol has demonstrated superiority in women with EAI; both GnRH agonist and antagonist protocols are effective and can be offered, as stated in the 2022 ESHRE guideline [54]. Antagonist protocols shorten treatment and allow an agonist trigger in patients at risk of OHSS; agonist long protocols may provide synchronization in selected patients but require longer suppression. Mild stimulation uses lower gonadotropin doses and may reduce burden at the cost of fewer oocytes and a greater risk of cycle cancellation [95,96]. Protocol selection should be based on ovarian reserve, prior response, safety, treatment burden, and laboratory practice rather than endometriosis diagnosis alone.

Progestin-primed ovarian stimulation prevents a premature luteinizing hormone surge through oral progestin administration and requires a freeze-all strategy because the endometrium is not synchronized for fresh transfer. Randomized trials in broader IVF populations have reported oocyte and pregnancy outcomes comparable with conventional protocols [97,98]. Evidence specific to endometriosis remains limited. Across available studies, no stimulation protocol has demonstrated universal superiority for EAI [87,88,99].

##### Fresh Versus Frozen Embryo Transfer

Frozen embryo transfer (FET) is clearly indicated when fresh transfer would be unsafe or biologically inappropriate, including substantial OHSS risk, progestin-primed stimulation, premature progesterone elevation, or a need for preimplantation genetic testing. Universal freeze-all, however, is not inherently superior. A cohort of non-PGT cycles reported similar pregnancy and live-birth outcomes after fresh and frozen transfer across age groups [100], and a randomized trial in women with a low prognosis found a higher live-birth rate after fresh transfer [101]. These data are not specific to endometriosis but illustrate the importance of prognosis and treatment context.

Meta-analyses and retrospective studies in women with endometriosis have reported higher implantation, clinical pregnancy, or live-birth rates after FET than after fresh transfer [102,103,104]. These findings are vulnerable to selection bias, differences in stimulation response and embryo availability, and confounding by indication. A 2025 meta-analysis reported an overall benefit of freeze-all in women with endometriosis, whereas no corresponding benefit was observed among women with adenomyosis [104], which emphasizes the need to distinguish these two conditions. The evidence supports individualized use of FET, but not routine freeze-all for every patient with endometriosis.

##### Intracytoplasmic Sperm Injection

Intracytoplasmic sperm injection (ICSI) is indicated for severe male-factor infertility, use of surgically retrieved sperm, or selected cases with prior total or near-total fertilization failure. In non-male-factor infertility, ICSI can reduce the risk of total fertilization failure but has not consistently improved cumulative live birth compared with conventional insemination [105,106]. Endometriosis alone is not an indication for routine ICSI. Laboratory history, semen quality, oocyte number, and the planned use of genetic testing should guide the insemination method.

### 7.5. Practical Treatment Framework

Based on the clinical considerations discussed above, the management of EAI should be individualized according to patient age, disease severity, ovarian reserve, tubal condition, and other fertility-associated factors. A practical treatment framework integrating these considerations is summarized in Table 3. 

## 8. Research Priorities and Limitations of the Evidence

The literature on EAI is limited by heterogeneous diagnostic criteria, incomplete phenotyping, variable inclusion of adenomyosis, previous surgery, and differences in ART practice. Many studies report clinical pregnancy rather than cumulative live birth and do not account adequately for age, ovarian reserve, embryo quality, male factor, or treatment selection. Molecular studies frequently analyze small tissue samples from surgical populations and may not distinguish eutopic endometrium, superficial lesions, endometrioma, and deep disease. These limitations can create apparent contradictions when pathway activity is compared across tissue compartments. They also limit the translation of mechanistic findings into clinically useful biomarkers, treatment-selection tools, or fertility-directed interventions.

Future studies should use standardized phenotypes, prespecified reproductive outcomes, and sufficient follow-up to capture cumulative live birth, obstetric safety, symptom burden, time to pregnancy, and patient-reported outcomes. Mechanistic trials should demonstrate target engagement, reproductive safety, and clinically meaningful fertility outcomes rather than extrapolating therapeutic efficacy from lesion size or inflammatory biomarkers alone. Comparative studies are particularly needed for fertility preservation before ovarian surgery, surgery versus direct IVF in well-defined subgroups, stimulation and transfer strategies, and the management of coexisting adenomyosis. Integration of imaging, clinical variables, and molecular data may eventually support useful prediction models, but external validation and transparent reporting are essential.

Regarding the quality of this review, we aimed to provide a comprehensive narrative review through a structured literature search, predefined review domains, and consideration of study design and clinical relevance. However, as the selection and discussion of individual studies involved author judgment, potential selection bias cannot be completely excluded. Nevertheless, the structured approach and evidence-based interpretation provide a comprehensive overview of current knowledge on EAI and its clinical management.

## 9. Conclusions

Endometriosis-associated infertility results from interacting anatomical, ovarian, inflammatory, immune, and endometrial mechanisms. The relative contribution of each pathway varies among patients and is not captured adequately by rASRM stage alone. Adenomyosis should be recognized as a distinct coexisting disorder that may further influence uterine function and pregnancy outcomes.

Clinical management should separate symptom-directed ovarian suppression from fertility-enhancing treatment. Surgery is valuable when it relieves clinically important symptoms, restores anatomy, treats organ-threatening disease, resolves diagnostic concern, or creates a realistic opportunity for natural conception; it should not be performed reflexively before ART, particularly for ovarian endometrioma. Stimulated IUI is reasonable for selected patients with mild disease and patent tubes, whereas IVF is favored when age, ovarian reserve, advanced disease, tubal pathology, male factor, or prior treatment failure makes delay disadvantageous. No single ovarian-stimulation or embryo-transfer strategy is optimal for all women with endometriosis, and ICSI is not indicated solely because endometriosis is present.

Overall, the clinical translation of EAI mechanisms remains uneven. Anatomical distortion, tubal dysfunction, and ovarian reserve impairment already inform fertility assessment and treatment selection, whereas inflammatory, immune, oxidative, endometrial, genetic, and epigenetic abnormalities remain largely non-actionable in current fertility practice. Emerging anti-angiogenic, immunomodulatory, antioxidant, stem-cell, and genetic or epigenetic approaches remain investigational. Distinguishing established clinical implications from biologically plausible but clinically unvalidated mechanisms may help prevent premature translation of molecular findings while identifying priorities for future fertility-directed research.

## Figures and Tables

**Table 1:** Revised American Society for Reproductive Medicine (rASRM) staging system for endometriosis. The rASRM score is based on the intraoperative assessment of endometriotic implants, ovarian involvement, posterior cul-de-sac obliteration, and ovarian and tubal adhesions. Disease is classified as Stage I (minimal, 1–5 points), Stage II (mild, 6–15 points), Stage III (moderate, 16–40 points), or Stage IV (severe, >40 points). The rASRM stage does not directly predict fertility outcomes [37].

INTRAOPERATIVE LAPAROSCOPIC ASSESSMENT							
• Score each site based on the largest lesion or extent observed. • Total rASRM score = Peritoneum + Ovary (endometrioma) + Posterior cul-de-sac obliteration + Ovarian adhesions + Tubal adhesions (0–176).							
**1 PERITONEUM (implants)**				**2 OVARY (endometrioma)**			
		**Lesion Size**	**Score**	**Lesion Size**		**Right Ovary** **Score**	**Left Ovary** **Score**
Superficial		<1 cm	1	Superficial	<1 cm	1	1
		1–3 cm	2		1–3 cm	2	2
		>3 cm	4		>3 cm	4	4
Deep		<1 cm	2	Deep	<1 cm	4	4
		1–3 cm	4		1–3 cm	16	16
		>3 cm	6		>3 cm	20	20
**Score (0–6):**			________	**Score (0–40):**			________
**3 POSTERIOR CUL-DE-SAC OBLITERATION (Pouch of Douglas)**							
Degree of Obliteration							
Partial				Complete			
4				40			
**Score (0 or 4 or 40): __________________**							
**4 ADHESIONS—OVARY** **(ovarian adhesions)**				**5 ADHESIONS—TUBE** **(tubal adhesions)**			
	**Extent of Adhesions**	**Right Ovary** **Score**	**Left Ovary** **Score**		**Extent of Adhesions**	**Right Tube** **Score**	**Left Tube** **Score**
Filmy	<1/3 enclosure	1	1	Filmy	<1/3 enclosure	1	1
	1/3–2/3 enclosure	2	2		1/3–2/3 enclosure	2	2
	>2/3 enclosure	4	4		>2/3 enclosure	4	4
Dense	<1/3 enclosure	4	4	Dense	<1/3 enclosure	4	4
	1/3–2/3 enclosure	8	8		1/3–2/3 enclosure	8	8
	>2/3 enclosure	16	16		>2/3 enclosure	16	16
**Score (0–32):**			________	**Score (0–64):**			________
**rASRM STAGE CLASSIFICATION**							
**STAGE I** **(Minimal)** **1–5 points**		**STAGE II** **(Mild)** **6–15 points**		**STAGE III** **(Moderate)** **16–40 points**		**STAGE IV** **(Severe)** **>40 points**	
• In the tube section, scores apply only when the fimbriated end of the fallopian tube is completely enclosed. • Superficial implants are red, red-pink, flame-like, vesicular, clear vesicles, white, or atypical lesions. • Percentages refer to the proportion of total involvement. • Maximum total score: 176 (Minimal disease = low score; Extensive disease = high score).							

**Table 2:** Endometriosis Fertility Index (EFI). The EFI combines historical factors (age, duration of infertility, and prior pregnancy) with surgical factors, including the least-function score and rASRM scores, to estimate the probability of non-IVF pregnancy after endometriosis surgery. The least-function score reflects the functional status of the fallopian tubes, fimbriae, and ovaries at the conclusion of surgery [35].

1. LEAST FUNCTION (LF) SCORE AT CONCLUSION OF SURGERY					
LF SCORE GUIDE		STRUCTURE	LEFT		RIGHT
**4**	Normal	**Fallopian Tube**	________________		________________
**3**	Mild Dysfunction	**Fimbria**	________________		________________
**2**	Moderate Dysfunction	**Ovary**	________________		________________
**1**	Severe Dysfunction	**LF SCORE** **COMPUTATION**	**Lowest score (Left) + Lowest score (Right)** = **LF score**		
**0**	Absent or Nonfunctional				
Find the lowest score on each side (Left and Right) for the three structures listed. If an ovary is not present on one side, determine the score by doubling the ovary score from the opposite side.					
**2. ENDOMETRIOSIS FERTILITY INDEX (EFI)**					
**HISTORICAL FACTORS**			**SURGICAL FACTORS**		
**FACTOR**	**CRITERIA**	**POINTS**	**FACTOR**	**CRITERIA**	**POINTS**
**Age**	≤35 years	2	**LF Score**	7–8 (high)	3
	36–39 years	1		4–6 (moderate)	2
	≥40 years	0		1–3 (low)	0
**Years** **Infertile**	≤3 years	2	**AFS Endometriosis** **Lesion Score**	<16	1
	>3 years	0		≥16	0
**Prior** **Pregnancy**	Yes	1	**AFS** **Total Score**	<71	1
	No	0		≥71	0
**TOTAL HISTORICAL FACTORS**		________	**TOTAL SURGICAL FACTORS**		________
**EFI score = Total historical-factor points + Total surgical-factor points (maximum score: 10).**					

Abbreviations: AFS, American Fertility Society; EFI, Endometriosis Fertility Index; LF, least function.

**Table 3:** Practical decision framework for endometriosis-associated infertility. Treatment should be individualized; the table is not a substitute for complete couple-based evaluation or shared decision-making. Abbreviations: AMH, anti-Müllerian hormone; EFI, Endometriosis Fertility Index; IUI, intrauterine insemination; IVF, in vitro fertilization.

Clinical Context	Reasonable First Approach	Important Cautions	Evidence Basis
Young patient, short infertility duration, rASRM stage I/II disease, patent tubes, favorable semen findings, postoperative patients with favorable EFI	Expectant management after complete evaluation or stimulated IUI	Reassess promptly if age, ovarian reserve, or duration makes delay consequential	ESHRE 2022 guideline Weak recommendation
Symptomatic endometrioma with atypia, rapid growth, inaccessible follicles, or major pain	Individualized fertility-preserving surgery	Counsel regarding AMH decline, bilateral disease, repeat surgery, and fertility preservation	ESHRE 2022 guideline Good practice point
Asymptomatic typical endometrioma before ART	Proceed to ART without routine surgery	Do not operate solely to improve live birth; review follicle access and malignancy risk	ESHRE 2022 guideline Strong recommendation
Severe hydrosalpinx	Salpingectomy or proximal occlusion followed by IVF	Balance surgical difficulty and ovarian blood supply; discuss risk of ectopic pregnancy	Supported by publications [82,83]
Advanced age, diminished reserve, low EFI, severe tubal disease, significant male factor, or failed IUI	IVF without unnecessary delay	Select stimulation and transfer strategy according to prognosis and safety	Supported by publications [92]
Pain requiring suppression while pregnancy is deferred	Combined hormonal contraceptive, progestin, GnRH analogue, or another guideline-supported pain treatment	Clarify that hormonal suppression is intended for symptom control rather than improving natural fertility	ESHRE 2022 guideline Strong recommendation
Recurrent implantation failure or suspected immune dysfunction	Reassess embryo, uterine, laboratory, and general clinical factors	Immunotherapy, microbiome treatment, or other unproven add-ons are not routinely recommended	Most emerging therapies remain preclinical, with limited evidence supporting their clinical use

## Data Availability

No new data were created or analyzed in this study. Data sharing is not applicable to this article.

## Supplementary Materials

The following supporting information can be downloaded at: https://www.mdpi.com/article/10.3390/ijms27209040/s1.

## References

[1] Chen Y., Waseem S., Luo L. (2025). Advances in the diagnosis and management of endometriosis: A comprehensive review. Pathol. Res. Pract..

[2] As-Sanie S., Mackenzie S.C., Morrison L., Schrepf A., Zondervan K.T., Horne A.W., Missmer S.A. (2025). Endometriosis. JAMA.

[3] Bulletti C., Coccia M.E., Battistoni S., Borini A. (2010). Endometriosis and infertility. J. Assist. Reprod. Genet..

[4] Sexual and Reproductive Health and Research (SRH) Infertility Prevalence Estimates, 1990–2021. https://www.who.int/publications/i/item/978920068315.

[5] Sallée C., Margueritte F., Marquet P., Piver P., Aubard Y., Lavoué V., Dion L., Gauthier T. (2022). Uterine Factor Infertility, a Systematic Review. J. Clin. Med..

[6] Allaire C., Bedaiwy M.A., Yong P.J. (2023). Diagnosis and management of endometriosis. Can. Med. Assoc. J..

[7] Lamceva J., Uljanovs R., Strumfa I. (2023). The main theories on the pathogenesis of endometriosis. Int. J. Mol. Sci..

[8] Mariadas H., Chen J.-H., Chen K.-H. (2025). The molecular and cellular mechanisms of endometriosis: From basic pathophysiology to clinical implications. Int. J. Mol. Sci..

[9] Bulun S.E. (2022). Endometriosis caused by retrograde menstruation: Now demonstrated by DNA evidence. Fertil. Steril..

[10] Matsuura K., Ohtake H., Katabuchi H., Okamura H. (1999). Coelomic Metaplasia Theory of Endometriosis: Evidence from in vivo Studies and an in vitro Experimental Model. Gynecol. Obstet. Investig..

[11] Maruyama T. (2022). A revised stem cell theory for the pathogenesis of endometriosis. J. Pers. Med..

[12] Koninckx P.R., Ussia A., Adamyan L., Wattiez A., Gomel V., Martin D.C. (2018). Pathogenesis of endometriosis: The genetic/epigenetic theory. Fertil. Steril..

[13] Chiorean D., Mitranovici M.-I., Toru H., Cotoi T., Tomuț A., Turdean S., Cotoi O. (2023). New Insights into Genetics of Endometriosis—A Comprehensive Literature Review. Diagnostics.

[14] Symons L.K., Miller J.E., Kay V.R., Marks R.M., Liblik K., Koti M., Tayade C. (2018). The Immunopathophysiology of Endometriosis. Trends Mol. Med..

[15] Dymanowska-Dyjak I., Terpiłowska B., Morawska-Michalska I., Michalski A., Polak G., Terpiłowski M., Rahnama-Hezavah M., Grywalska E. (2024). Immune dysregulation in endometriomas: Implications for inflammation. Int. J. Mol. Sci..

[16] Schrager S., Yogendran L., Marquez C.M., Sadowski E.A. (2022). Adenomyosis: Diagnosis and Management. Am. Fam. Physician.

[17] Pados G., Gordts S., Sorrentino F., Nisolle M., Nappi L., Daniilidis A. (2023). Adenomyosis and Infertility: A literature review. Medicina.

[18] Szubert M., Koziróg E., Olszak O., Krygier-Kurz K., Kazmierczak J., Wilczynski J. (2021). Adenomyosis and Infertility—Review of medical and surgical approaches. Int. J. Environ. Res. Public Health.

[19] Wang S., Duan H. (2023). The role of the junctional zone in the management of adenomyosis with infertility. Front. Endocrinol..

[20] Dai Y., Yuan Z., Fan W., Lin Z. (2025). Molecular mechanism of aberrant decidualization in adenomyosis leading to reduced endometrial receptivity. Front. Endocrinol..

[21] An M., Fu X., Meng X., Liu H., Ma Y., Li Y., Li Q., Chen J. (2024). PI3K/AKT signaling pathway associates with pyroptosis and inflammation in patients with endometriosis. J. Reprod. Immunol..

[22] Hu Y., Chen H., Jin L., Chi X., Zhao J., Cao Q. (2025). Hypomethylation of IL6ST promotes development of endometriosis by activating JAK2/STAT3 signaling pathway. PLoS ONE.

[23] Roberson E.C., Tran N.K., Godambe A.N., Mark H., Nguimtsop M., Rust T., Ung E., Barker L.J., Fitch R.D., Wallingford J.B. (2023). Hedgehog signaling is required for endometrial remodeling and myometrial homeostasis in the cycling mouse uterus. iScience.

[24] Zhou Y., Peng Y., Xia Q., Yan D., Zhang H., Zhang L., Chen Y., Zhao X., Li J. (2020). Decreased Indian hedgehog signaling activates autophagy in endometriosis and adenomyosis. Reproduction.

[25] Moawad G., Fruscalzo A., Youssef Y., Kheil M., Tawil T., Nehme J., Pirtea P., Guani B., Afaneh H., Ayoubi J.M. (2023). Adenomyosis: An Updated Review on Diagnosis and Classification. J. Clin. Med..

[26] Cunningham R.K., Horrow M.M., Smith R.J., Springer J. (2018). Adenomyosis: A Sonographic Diagnosis. Radiographics.

[27] Hoyle A.T., Puckett Y. (2023). Endometrioma.

[28] Nezhat F.R., Cathcart A.M., Nezhat C.H., Nezhat C.R. (2024). Pathophysiology and clinical implications of ovarian endometriomas. Obstet. Gynecol..

[29] Wu M.-H., Cheng Y.-C., Chang F.-M. (2008). Ultrasonographic Assessment of ovarian endometrioma. J. Med. Ultrasound.

[30] Imperiale L., Nisolle M., Noël J.-C., Fastrez M. (2023). Three types of endometriosis: Pathogenesis, diagnosis and treatment. State of the art. J. Clin. Med..

[31] Del Frate C., Girometti R., Pittino M., Del Frate G., Bazzocchi M., Zuiani C. (2006). Deep Retroperitoneal Pelvic Endometriosis: MR Imaging Appearance with Laparoscopic Correlation. Radiographics.

[32] Teng S.-W., Horng H.-C., Ho C.-H., Yen M.-S., Chao H.-T., Wang P.-H., Chang Y.-H., Chang Y., Chao K.-C., Chen Y.-J. (2016). Women with endometriosis have higher comorbidities: Analysis of domestic data in Taiwan. J. Chin. Med. Assoc..

[33] Practice Committee of the American Society for Reproductive Medicine (2021). Fertility evaluation of infertile women: A committee opinion. Fertil. Steril..

[34] Xue Q., Li X., Zeng C., Zhou Y.-F., Yang H.-X., Shang J., Zhu S.-N. (2017). Endometriosis Fertility Index for Predicting Pregnancy after Endometriosis Surgery. Chin. Med. J..

[35] Adamson G.D., Pasta D.J. (2010). Endometriosis fertility index: The new, validated endometriosis staging system. Fertil. Steril..

[36] Marchetti M., Noventa M., Panizzolo E., Pianon V., Tamagnini M., Bigardi S., Saccardi C., Tozzi R., Spagnol G. (2024). An Ultrasound-Based Preoperative Evaluation of the Endometriosis Fertility Index: A Further Step towards Personalized Treatment. J. Clin. Med..

[37] American Society for Reproductive Medicine (1997). Revised American Society for Reproductive Medicine classification of endometriosis: 1996. Fertil. Steril..

[38] Carson S.A., Kallen A.N. (2021). Diagnosis and management of infertility. JAMA.

[39] Phillips K., Olanrewaju R.A., Omole F. (2023). Infertility: Evaluation and management. Am. Fam. Physician.

[40] Özcan P., Varlı B., Sarıdoğan E., Oral E., Mabrouk M., Usta T., Constantin A.S. (2025). Mechanisms of Endometrioma-Mediated Ovarian Damage: Myths and Facts. J. Clin. Med..

[41] Hanege B.Y., Çekıç S.G., Ata B. Endometrioma and Ovarian Reserve: Effects of Endometriomata per se and Its Surgical Treatment on the Ovarian Reserve. https://pmc.ncbi.nlm.nih.gov/articles/PMC6897522/.

[42] Lu R., Jiao W., Li X., Zeng C., Shang J., Xue Q. (2025). Ovarian endometriosis negatively impacts pregnancy outcomes in young infertile women undergoing IVF/ICSI treatment. Eur. J. Med. Res..

[43] Ambildhuke K., Pajai S., Chimegave A., Mundhada R., Kabra P. (2022). A Review of Tubal Factors Affecting Fertility and its Management. Cureus.

[44] The Practice Committee of the American Society for Reproductive Medicine (2021). Role of tubal surgery in the era of assisted reproductive technology: A committee opinion. Fertil. Steril..

[45] Qi Q., Li Y., Chen Z., Luo Z., Zhou T., Zhou J., Zhang Y., Chen S., Wang L. (2025). Update on the pathogenesis of endometriosis-related infertility based on contemporary evidence. Front. Endocrinol..

[46] Approbato F.C., Approbato M.S., Rezende D.F., Da Silva T.M., De Lima Y.A.R., Benetti B.B.D.C. (2019). Endometriosis III and IV as a risk factor for tubal obstruction in infertile women. JBRA.

[47] Demirel E., Ali A., Seraji S., Khullar P., Nezhat F.R. (2024). Incidence of fallopian tube endometriosis and its association with endometriomas, hydrosalpinx, and peritubal adhesions: A prospective cohort study. Fertil. Steril..

[48] Pirtea P., De Ziegler D., Ayoubi J.M. (2023). Endometrial receptivity in adenomyosis and/or endometriosis. Fertil. Steril..

[49] Ghobrial S., Ott J., Parry J.P. (2023). An Overview of postoperative intraabdominal adhesions and their role on female infertility: A narrative review. J. Clin. Med..

[50] Lin J., Yang Z., Tan T., Wang L., Xiao Z., Chen J. (2025). Effect of pelvic adhesions on reproductive outcomes following high-intensity focused ultrasound in patients with adenomyosis. Int. J. Gynecol. Obstet..

[51] Seleș L.G., Murvai V.R., Maghiar L., Bodog A., Huniadi A. (2026). Endometriosis uncovered: From chronic inflammation to reproductive dysfunction and impaired ART outcomes. Medicina.

[52] Bouic P.J. (2023). Endometriosis and infertility: The hidden link between endometritis, hormonal imbalances and immune dysfunctions preventing implantation!. JBRA.

[53] Ou Y., Wang H., Zhou C., Chen Y., Lyu J., Feng M., Huang X. (2025). Endometriosis-associated infertility: Multi-omics insights into pathogenesis and precision therapeutics. Front. Endocrinol..

[54] Becker C.M., Bokor A., Heikinheimo O., Horne A., Jansen F., Kiesel L., King K., Kvaskoff M., Nap A., Petersen K. (2022). ESHRE guideline: Endometriosis. Hum. Reprod. Open.

[55] Pados G., Gordts S., Sorrentino F., Nisolle M., Nappi L., Daniilidis A., Elbasueny B., Geerts M., Yang E.C., Allaire C. (2022). Medical treatments of endometriosis: A review. Reprod. Dev. Med..

[56] Barton B.E., Erickson J.A., Allred S.I., Jeffries J.M., Stephens K.K., Hunter M.I., Woodall K.A., Winuthayanon W. (2023). Reversible female contraceptives: Historical, current, and future perspectives. Biol. Reprod..

[57] Khalifa E., Mohammad H., Abdullah A., Abdel-Rasheed M., Khairy M., Hosni M. (2021). Role of suppression of endometriosis with progestins before IVF-ET: A non-inferiority randomized controlled trial. BMC Pregnancy Childbirth.

[58] Li D., Zheng L., Zhang X., Wang W., Zhang J., Fu L. (2026). Efficacy of different pretreatments in IVF outcomes in patients with endometriosis: A systematic review and network meta-analysis. Sci. Rep..

[59] Steinmann M., Anthon C., Schwartz A.S.K., Mackens S., Blockeel C., Kalaitzopoulos D.R., Vidal A. (2025). GnRH agonist pretreatment prior to frozen embryo transfer in women with adenomyosis: A systematic review and meta-analysis. Reprod. Biomed. Online.

[60] Taylor H., Li H.J., Carson S., Flores V., Pal L., Robbins J., Santoro N.F., Segars J.H., Seifer D., Huang H. (2022). Pre-IVF treatment with a GnRH antagonist in women with endometriosis (PREGNANT): Study protocol for a prospective, double-blind, placebo-controlled trial. BMJ Open.

[61] Hashim H.A. (2016). Aromatase Inhibitors for Endometriosis-Associated Infertility; Do we have sufficient evidence?. Int. J. Fertil. Steril..

[62] Chung M.S., Han S.J. (2022). Endometriosis-Associated angiogenesis and anti-angiogenic therapy for endometriosis. Front. Glob. Women’s Health.

[63] Kim Y.S., Kim Y.J., Kim M.J., Lee S.J., Kwon H., Lee J.H. (2020). Novel Medicine for endometriosis and its therapeutic effect in a mouse model. Biomedicines.

[64] Mehakar S., More A., Dutta S., Jadhav R., Chaudhari N., Shaikh R. (2023). Case Report: Implication of antiangiogenic therapy (bevacizumab) along with dichloroacetate therapy on endometriosis patient seeking infertility treatment. F1000Research.

[65] Hammond O., El-Sheikh O., Saad R.M., Shetty P., Papakonstantinou E., Kastora S.L. (2026). Targeting angiogenesis in endometriosis: A systematic review and network meta-analysis of VEGF-directed pharmacotherapies. Front. Reprod. Health.

[66] Chen Q., Wang J., Ding X., Zhang Q., Duan P. (2024). Emerging strategies for the treatment of endometriosis. Biomed. Technol..

[67] Li W., Lin A., Qi L., Lv X., Yan S., Xue J., Mu N. (2023). Immunotherapy: A promising novel endometriosis therapy. Front. Immunol..

[68] Liu M., Li Y., Yuan Y., Jiang M., Yin P., Yang D. (2024). Peri-implantation treatment with TNF-α inhibitor for endometriosis and/or adenomyosis women undergoing frozen-thawed embryo transfer: A retrospective cohort study. J. Reprod. Immunol..

[69] Dymanowska-Dyjak I., Frankowska K., Abramiuk M., Polak G. (2024). Oxidative Imbalance in Endometriosis-Related Infertility—The therapeutic role of antioxidants. Int. J. Mol. Sci..

[70] Zhong Y., Qiao X., Huang X., Li Y., Wang R., Liang J., Liu T., Bo W., Lai H., Huang W. (2025). The effects of antioxidant supplementation on pain, oxidative stress markers, and clinical pregnancy rate in women with endometriosis: A systematic review and meta-analysis of randomized controlled trials. Front. Med..

[71] Suárez-Carrasco I., De Pedro M.Á., Pulido M., Álvarez V., Sánchez-Margallo F.M., López E. (2026). Stem cell therapy-based approaches in experimental endometriosis: A systematic review. Reprod. Biol. Endocrinol..

[72] Huang S.J., Huang C.-Y., Huang Y.-H., Cheng J.-H., Yu Y.-C., Lai J.-C., Hung Y.-P., Chang C.-C., Shiu L.-Y. (2023). A novel therapeutic approach for endometriosis using adipose-derived stem cell-derived conditioned medium—A new hope for endometriotic patients in improving fertility. Front. Endocrinol..

[73] Bischiniotis S., Mikos T., Grimbizis G.F. (2024). Surgical treatment of adenomyosis. Curr. Obstet. Gynecol. Rep..

[74] Dessouky R., Gamil S.A., Nada M.G., Mousa R., Libda Y. (2019). Management of uterine adenomyosis: Current trends and uterine artery embolization as a potential alternative to hysterectomy. Insights Imaging.

[75] Capezzuoli T., Toscano F., Ceccaroni M., Roviglione G., Stepniewska A., Fambrini M., Vannuccini S., Petraglia F. (2024). Conservative surgical treatment for adenomyosis: New options for looking beyond uterus removal. Best Pract. Res. Clin. Obstet. Gynaecol..

[76] Moawad G., Youssef Y., Fruscalzo A., Faysal H., Merida M., Pirtea P., Guani B., Ayoubi J.M., Feki A. (2024). The impact of conservative surgical treatment of adenomyosis on fertility and perinatal outcomes. J. Clin. Med..

[77] Grammatis A.L., Lazaridis A., Becker C.M., Saridogan E. (2024). Surgical techniques for ovarian endometriosis. Obstet. Gynaecol..

[78] Muzii L., Galati G., Mattei G., Chinè A., Perniola G., Di Donato V., Di Tucci C., Palaia I. (2023). Expectant, medical, and surgical management of ovarian endometriomas. J. Clin. Med..

[79] Younis J.S., Taylor H.S. (2024). The impact of ovarian endometrioma and endometriotic cystectomy on anti-Müllerian hormone, and antral follicle count: A contemporary critical appraisal of systematic reviews. Front. Endocrinol..

[80] AlKudmani B., Gat I., Buell D., Salman J., Zohni K., Librach C., Sharma P. (2017). In Vitro Fertilization Success Rates after Surgically Treated Endometriosis and Effect of Time Interval between Surgery and In Vitro Fertilization. J. Minim. Invasive Gynecol..

[81] Mayrhofer D., Parry J.P., Hager M., Beitl K., Kurz C., Ott J. (2022). Are the Stage and the Incidental Finding of Endometriosis Associated with Fallopian Tube Occlusion? A Retrospective Cohort Study on Laparoscopic Chromopertubation in Infertile Women. J. Clin. Med..

[82] Tamblyn J., Jeve Y. (2022). Surgical management of tubal disease and infertility. Obstet. Gynaecol. Reprod. Med..

[83] World Health Organization (2025). Treatment of infertility due to tubal disease. Guideline for the Prevention, Diagnosis and Treatment of Infertility.

[84] Tanaka Y.O., Ota Y., Tamura A., Kobori H., Kitai S., Yanai S., Shiokawa R., Takubo R., Yamamoto M., Okada N. (2025). MRI as a promising tool for adhesion assessment in endometriosis: A feasibility study with laparoscopic correlation. Eur. J. Obstet. Gynecol. Reprod. Biol..

[85] Somigliana E., Vigano P., Benaglia L., Busnelli A., Vercellini P., Fedele L. (2012). Adhesion Prevention in Endometriosis: A neglected critical challenge. J. Minim. Invasive Gynecol..

[86] Krämer B., Andress J., Neis F., Hoffmann S., Brucker S., Kommoss S., Höller A. (2021). Adhesion prevention after endometriosis surgery—Results of a randomized, controlled clinical trial with second-look laparoscopy. Langenbeck’s Arch. Surg..

[87] Gniadek M., Porubenska A., Pełka K., Rzepkowska Z., Florjański J. (2026). Endometriosis-Associated Infertility: A review of pathophysiological mechanisms and current treatment strategies. J. Clin. Med..

[88] Elizur S.E., Mostafa J., Berkowitz E., Orvieto R. (2025). Endometriosis and infertility: Pathophysiology, treatment strategies, and reproductive outcomes. Arch. Gynecol. Obstet..

[89] Allahbadia G.N. (2017). Intrauterine Insemination: Fundamentals Revisited. J. Obstet. Gynecol. India.

[90] Chronopoulou E., Gaetano-Gil A., Shaikh S., Raperport C., Wattar B.H.A., Ruiz-Calvo G., Zamora J., Bhide P. (2024). Optimizing intrauterine insemination: A systematic review and meta-analysis of the effectiveness and safety of clinical treatment add-ons. Acta Obstet. Gynecol. Scand..

[91] Van Der Houwen L.E.E., Schreurs A.M.F., Schats R., Heymans M.W., Lambalk C.B., Hompes P.G.A., Mijatovic V. (2014). Efficacy and safety of intrauterine insemination in patients with moderate-to-severe endometriosis. Reprod. Biomed. Online.

[92] Wang J., Sauer M.V. (2006). In vitro fertilization (IVF): A review of 3 decades of clinical innovation and technological advancement. Ther. Clin. Risk Manag..

[93] Bentov Y., Schenker J. (2025). IVF and pregnancy outcomes: The triumphs, challenges, and unanswered questions. J. Ovarian Res..

[94] Mancuso F., Singh M., Shanks A.L. (2026). In Vitro Fertilization.

[95] Shrestha D., La X., Feng H.L. (2015). Comparison of different stimulation protocols used in in vitro fertilization: A review. Ann. Transl. Med..

[96] Alper M.M., Fauser B.C. (2017). Ovarian stimulation protocols for IVF: Is more better than less?. Reprod. Biomed. Online.

[97] Xi Q., Tao Y., Qiu M., Wang Y., Kuang Y. (2020). Comparison Between PPOS and GnRHa-Long Protocol in Clinical Outcome with the First IVF/ICSI Cycle: A Randomized Clinical Trial. Clin. Epidemiol..

[98] Meseci E., Okten S.B., Bilgen S., Ozveri H., Ocal N., Ozdemir B., Fıcıcıoglu C. (2025). Progestin primed ovarian stimulation yields comparable outcomes to the GnRH antagonist protocol for controlled ovarian hyperstimulation. Sci. Rep..

[99] Latif S., Khanjani S., Saridogan E. (2024). Endometriosis and in vitro fertilization. Medicina.

[100] Pavlovic Z.J., Smotrich G.E., New E.P., Jahandideh S., Devine K., Imudia A.N., Plosker S. (2024). Fresh vs. frozen: Pregnancy outcomes and treatment efficacy between fresh embryo transfer vs. untested freeze-all cycles. F&S Rep..

[101] Wei D., Sun Y., Zhao H., Yan J., Zhou H., Gong F., Zhang A., Wang Z., Jin L., Bao H. (2025). Frozen versus fresh embryo transfer in women with low prognosis for in vitro fertilisation treatment: Pragmatic, multicentre, randomised controlled trial. BMJ.

[102] Chang Y., Shen M., Wang S., Li X., Duan H. (2022). Association of embryo transfer type with infertility in endometriosis: A systematic review and meta-analysis. J. Assist. Reprod. Genet..

[103] Shoukry A., Gazeirly W.S.E., Khattab M.A., Abdelmoneim H.M.A. (2024). Reproductive outcomes in women with advanced endometriosis in fresh versus frozen embryo transfer cycles. Middle East Fertil. Soc. J..

[104] Han Y., Liu C., Liu D., Wu L., Huang W. (2025). Pregnancy outcomes in freeze-all versus fresh embryo transfer cycles of women with adenomyosis and endometriosis: A systemic review and meta-analysis. Front. Endocrinol..

[105] Iwamoto A., Summers K.M., Sparks A., Mancuso A.C. (2024). Intracytoplasmic sperm injection versus conventional in vitro fertilization in unexplained infertility. F&S Rep..

[106] Berntsen S., Zedeler A., Nøhr B., Petersen M.R., Grøndahl M.L., Andersen L.F., Løssl K., Løkkegaard E., Englund A.L., Gabrielsen A.V. (2025). IVF versus ICSI in patients without severe male factor infertility: A randomized clinical trial. Nat. Med..

