# Supplementary material for: Endometriosis-Associated Infertility: Molecular Mechanisms and Evidence-Based Clinical Management
Source: Int J Mol Sci. 2026 Oct 12;27(20):9040. doi: 10.3390/ijms27209040 (PMC13705522; doi:10.3390/ijms27209040)
Supplement: Supplementary file 1 [file ijms-27-09040-s001.zip › ijms-4511402-supplementary.pdf]

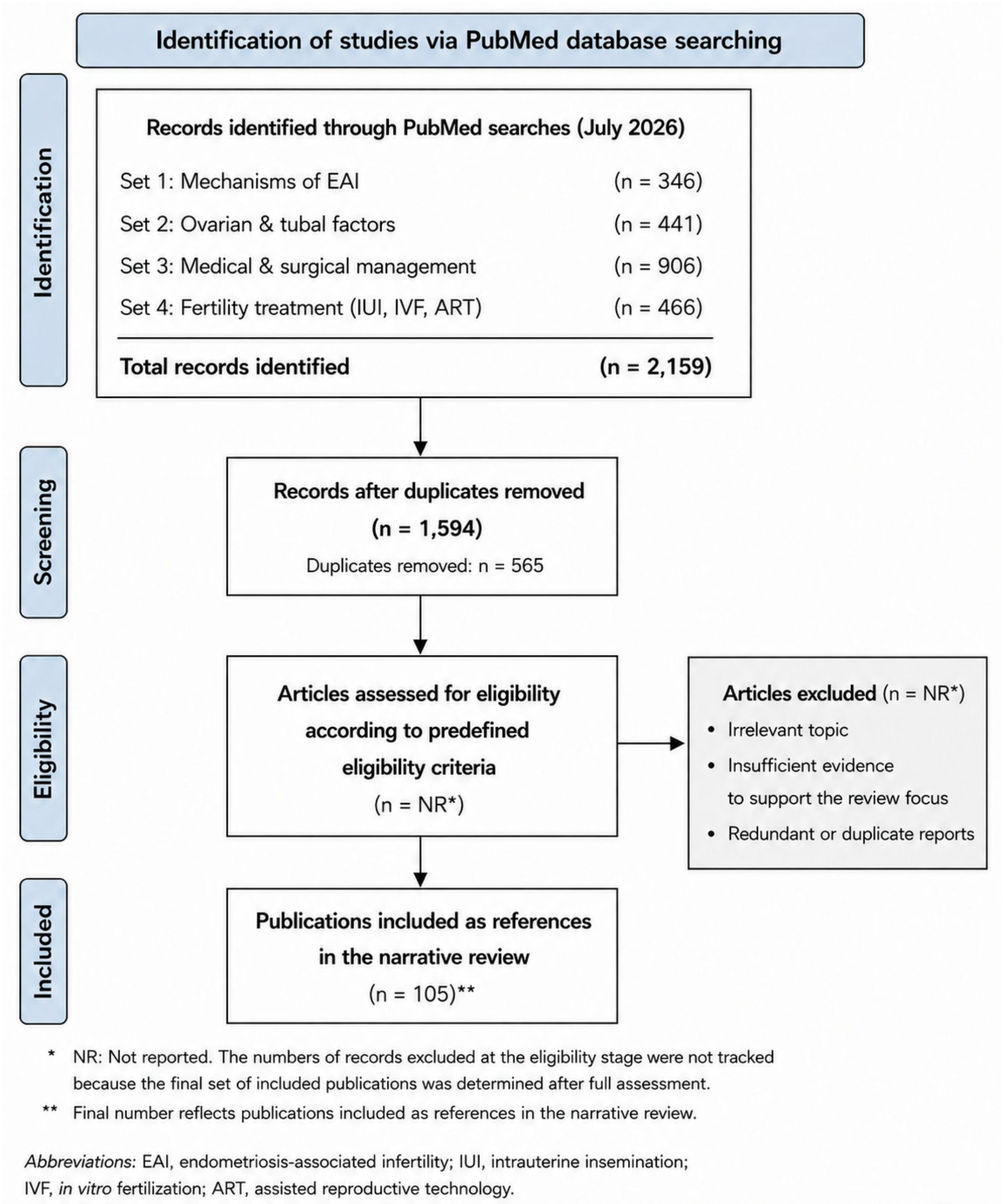

Supplementary Figure S1. The flowchart of database searching, screening, eligibility assessment, and inclusion of publications in the review.
